# Transcranial Direct Current Stimulation of Primary Motor Cortex over Multiple Days Improves Motor Learning of a Complex Overhand Throwing Task

**DOI:** 10.3390/brainsci13101441

**Published:** 2023-10-10

**Authors:** Milan Pantovic, Lidio Lima de Albuquerque, Sierra Mastrantonio, Austin S. Pomerantz, Erik W. Wilkins, Zachary A. Riley, Mark A. Guadagnoli, Brach Poston

**Affiliations:** 1 Health and Human Performance Department, Utah Tech University, St. George, UT 84770, USA; milan.pantovic@utahtech.edu; 2 School of Health and Applied Human Sciences, University of North Carolina-Wilmington, Wilmington, NC 28403, USA; limadeal@uncw.edu; 3 School of Medicine, University of Nevada-Las Vegas, Las Vegas, NV 89154, USA; kreamer-h@unlv.nevada.edu (S.M.); pomeran7@unlv.nevada.edu (A.S.P.); mark.guadagnoli@unlv.edu (M.A.G.); 4 Department of Kinesiology and Nutrition Sciences, University of Nevada-Las Vegas, Las Vegas, NV 89154, USA; wilkie1@unlv.nevada.edu; 5 Department of Kinesiology, Indiana University Purdue University Indianapolis, Indianapolis, IN 46202, USA; zariley@iupui.edu

**Keywords:** motor skill, transcranial magnetic stimulation, transcranial direct current stimulation

## Abstract

Transcranial direct current stimulation (tDCS) applied to the primary motor cortex (M1) improves motor learning in relatively simple motor tasks performed with the hand and arm. However, it is unknown if tDCS can improve motor learning in complex motor tasks involving whole-body coordination with significant endpoint accuracy requirements. The primary purpose was to determine the influence of tDCS on motor learning over multiple days in a complex over-hand throwing task. This study utilized a double-blind, randomized, SHAM-controlled, between-subjects experimental design. Forty-six young adults were allocated to either a tDCS group or a SHAM group and completed three experimental sessions on three consecutive days at the same time of day. Each experimental session was identical and consisted of overhand throwing trials to a target in a pre-test block, five practice blocks performed simultaneously with 20 min of tDCS, and a post-test block. Overhand throwing performance was quantified as the endpoint error. Transcranial magnetic stimulation was used to obtain motor-evoked potentials (MEPs) from the first dorsal interosseus muscle to quantify changes in M1 excitability due to tDCS. Endpoint error significantly decreased over the three days of practice in the tDCS group but not in the SHAM group. MEP amplitude significantly increased in the tDCS group, but the MEP increases were not associated with increases in motor learning. These findings indicate that tDCS applied over multiple days can improve motor learning in a complex motor tasks in healthy young adults.

## 1. Introduction

Transcranial direct current stimulation (tDCS) applied to the primary motor cortex (M1) has been shown in numerous studies to increase primary motor cortex excitability and improve motor skill learning in tasks performed with the hand and arm [1,2,3,4,5]. Most M1-tDCS studies have shown skill improvements of approximately 10–15% during or immediately after a single tDCS application when compared to the practice of a motor task alone in the same conditions [1]. Based on classic studies [6,7,8] that demonstrated increased primary motor cortex excitability following the practice of fine motor skills, it was originally thought that the primary motor cortex excitability increases elicited during and after application of anodal M1-tDCS could further enhance the normal use-dependent plasticity processes that occur with motor practice, leading to greater motor learning [1,9]. In addition, these ideas were also based on basic physiology studies that suggested that tDCS effects were due to increases in the subthreshold modulation of membrane potentials of neurons [10], modulations in GABAergic receptor activity [10], and long-term potentiation, as well as homeostatic metaplasticity type mechanisms that modify the strength of synaptic connections between neurons [1,11]. Accordingly, the most common strategy in tDCS motor skill studies has been to simultaneously apply M1-tDCS concurrent with motor practice [1,12,13].

The vast majority of studies that have applied tDCS to any brain region have involved only one 10 to 20 min practice session [1,14]. However, a few studies that have applied M1-tDCS for 3–5 consecutive days have been able to induce cumulative effects, leading to an approximate enhancement of 20–40% in total motor learning when compared to SHAM stimulation [12,13]. Both of these studies involved a sequential visuomotor isometric pinch grip task (SVIPT) of the thumb and index finger as the motor task. Interestingly, the greater gains in total motor learning in the tDCS groups in these studies compared to the SHAM were primarily due to consolidation processes that took place between sessions (also termed “offline” learning effects) as opposed to within-session (also termed “online” learning effects) skill gains, which was similar between groups [12,13]. Overall, the multi-session tDCS studies have particularly important implications for enhancing performance in various motor tasks due to the fact that the increases in motor skills accumulate to a much higher degree compared to single-session studies.

Although the findings of single- and multiple-day tDCS studies have been promising, almost all of them have utilized relatively simple fine motor tasks, such as finger sequences with four digits, two-dimensional arm reaching movements, various pinch grip tasks involving the thumb and index finger, hand/arm dexterity tasks, and single-joint visuomotor tracking tasks [1,15,16]. In addition, many of these are often laboratory tasks that were novel to the participants or performed in contexts different from those encountered in activities of daily living. Thus, it is relatively unknown if tDCS can enhance motor learning in complex tasks that involve multiple joints, whole-body coordination, and high accuracy requirements. While simple motor tasks are important as initial starting points in research, allow for very strict controlled experiments, and make simultaneous physiological measurements much easier to obtain, the investigation of complex motor tasks is needed to fully understand human movement control and motor learning [17,18,19]. Most importantly, complex motor tasks are much more relevant to the workplace, military, sports, and other real-world tasks involved in the activities of daily living. Taken together, these lines of reasoning imply that the viability of tDCS as an intervention to increase motor learning can only be determined through the examination of complex motor tasks.

The primary purpose was to examine the influence of tDCS on motor learning over multiple days in a complex overhand throwing task in young adults. To accomplish this, two groups of participants completed three practice sessions of overhand throws on consecutive days simultaneously with either tDCS or SHAM stimulation. Based on both single-session and multi-day tDCS studies that involved relatively simple motor tasks [1], it was predicted that tDCS applied to the primary motor cortex over three days would improve endpoint accuracy in a complex overhand throwing task. Specifically, it was expected that tDCS would lead to greater online learning, offline learning, and total motor learning compared to SHAM stimulation. The secondary purpose was to examine the association between tDCS-induced increases in cortical excitability and the magnitude of motor learning. Based on previous studies [20,21], it was anticipated that the increase in motor-evoked potential (MEP) amplitude following tDCS would be positively associated with the degree of motor learning exhibited by participants in the tDCS group.

## 2. Materials and Methods

### 2.1. Participants

**Figure 1:**
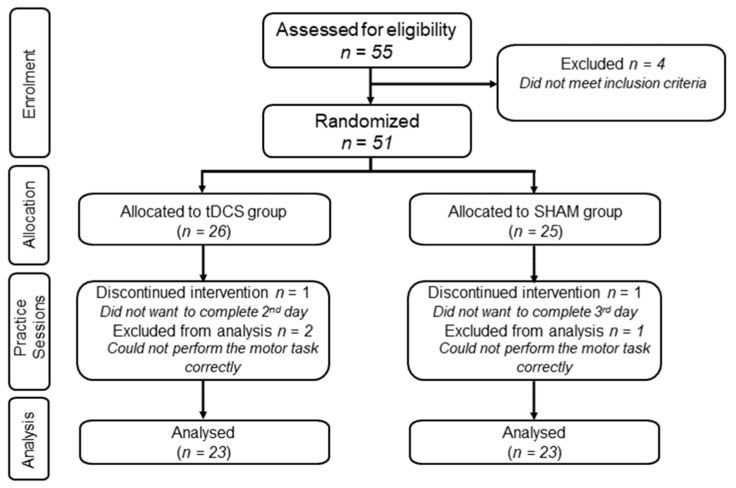
CONSORT flow diagram illustrating enrollment, allocation, practice sessions, and analysis portions of tracking participants over the course of the study.

**Figure 2:**
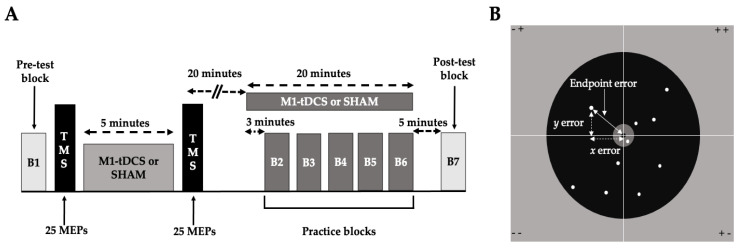
A schematic illustration of the major elements of the experimental paradigm. (**A**) Three identical consecutive daily experimental sessions were performed, but only one experimental session is shown for brevity and illustrative purposes. The protocol involved a pre-test block of overhand throws, a TMS testing protocol to determine the effects of tDCS on primary motor cortex excitability, five practice blocks of overhand throws performed simultaneous with 20 min of tDCS or SHAM stimulation, and a post-test block of overhand throws; (**B**) the quantification of endpoint error and a representative group of trials. Endpoints of the ball are depicted for a 10-trial block, as well as the *x* and *y* errors for a single trial that were used to calculate the endpoint error of the trial.

A total of 46 young adults completed the study (26 males and 20 females; mean age: 24.9 ± 3.4; range: 20–32 years). The tDCS group (mean age: 25.7 ± 3.6) and the SHAM group (mean age: 24.2 ± 3.1) both consisted of 13 males and 10 females. Figure 1 depicts the CONSORT flow diagram of the study. Briefly, 55 participants were assessed for eligibility, with 4 being excluded for not meeting the inclusion criteria. Thus, 51 were randomized and allocated to the two groups. A total of 5 participants did not complete the study, with 2 of them (1 per group) dropping out due to not wanting to complete all 3 sessions. In addition, 3 participants (2 tDCS group; 1 SHAM group) were dropped by the investigators as they could not perform the motor task correctly; they failed in a high number of trials to even hit the wall with the ball. Specifically, many throws either hit the ceiling or the floor, which would obviously preclude measuring the accuracy of the throw on the two-dimensional target on the wall for many trials (see Figure 2B and Section 2.5: Overhand Throwing Task) and render measures of motor learning meaningless.

Participants were all strongly right-handed based on the Edinburgh Handedness Inventory [22], with an average laterality index of 0.82 ± 0.19 (tDCS group: 0.81 ± 0.23; SHAM group: 0.83 ± 0.15). All participants threw with their right hand, had no known history of neurological disorders, did not report any psychiatric condition, and did not meet any exclusion criteria for non-invasive brain stimulation [23]. Potential participants who were currently engaged in a throwing sport on the recreational, collegiate, or professional level were excluded from this study. Participants provided written, informed consent before participating in the study. All experimental procedures were approved by the University of Nevada, Las Vegas Institutional Review Board and conducted according to the Declaration of Helsinki.

### 2.2. Experimental Design

This study utilized a double-blind, randomized, between-subjects, SHAM-controlled experimental design. Participants were allocated to either a tDCS or a SHAM stimulation group using a Research Randomizer (www.randomizer.org; accessed on 4 January 2018) by an investigator who was not involved in data collection. The participants came to the laboratory at the same time of day on three consecutive days and completed three identical experimental sessions, with each session lasting ~1.5–2 h. The one exception was that a familiarization involving a short instructional video and an overhand throwing demonstration was completed at the start of the first experimental session. The major experimental steps for a single experimental session are depicted in Figure 2A and were completed in the following order: (1) a pre-test block of overhand throwing trials (no stimulation); (2) transcranial magnetic stimulation (TMS) testing of the effect of tDCS on primary motor cortex excitability that collectively involved a TMS pre-test, five minutes of either tDCS or SHAM stimulation, and a TMS post-test; (3) practice blocks of overhand throwing conducted simultaneously with either tDCS or SHAM; and (4) a post-test block of overhand throwing trials (no stimulation). The finer methodological details aspects of each of the major steps are provided in the sections below. The investigators who conducted the experiments detailed below and analyzed the data were blind to the participant group assignments. Furthermore, the investigator who applied stimulation and operated the tDCS device did not partake in the other experimental procedures.

### 2.3. Experimental Procedures

#### 2.3.1. Pre-Test Block

The pre-test block of 10 overhand throwing trials was executed without simultaneous tDCS application to quantify baseline performance for the groups on day 1 before any stimulation had been applied. Accordingly, the pre-test blocks on days two and three were executed in the same manner and provided a baseline on those days that were not influenced by stimulation but could have been impacted by consolidation effects from the prior day. For all pre-test blocks, 10 trials per block were selected, as this was previously deemed to be adequate [24] for baseline data but did not overly impact subsequent overhand throwing performance curves in the practice blocks. Additionally, this ensured that the number of trials per block was the same as the post-test and practice blocks. The performance of the pre-test blocks without simultaneous tDCS application permitted the calculation of the relative roles of online and offline learning effects to the total motor learning (see Section 2.7).

#### 2.3.2. Transcranial Magnetic Stimulation Quantification of tDCS Effects on Primary Motor Cortex Excitability

TMS was performed with a Magstim 200^2^ that was connected to a double 70 mm remote control figure-of-eight coil that was arranged tangentially to the scalp with the handle pointing laterally and backward at a 45-degree angle relative to the midline. An investigator positioned the coil over the “motor hot spot” of the first dorsal interosseous (FDI) muscle of the left primary motor cortex to obtain MEPs in the FDI of the right (contralateral) hand [25]. Electromyographic (EMG) activity of the FDI was recorded with surface electrodes arranged in a belly tendon montage. Cambridge Electronic Design (CED; Cambridge, UK) hardware (1902 amplifiers, micro 1401 data acquisition interface) and software (Signal) were used to acquire and record all EMG signals. MEPs were evoked with single TMS pulses while subjects were seated with the forearm on a table, the wrist in a neutral position, the hand prone, the elbow flexed (~90 degrees), and the shoulder abducted to ~45 degrees. Most importantly, the FDI muscle was at rest during all MEP recordings, and participants were provided with EMG feedback on a computer monitor. One investigator further monitored the participant and EMG signals constantly to ensure that the FDI was at rest during TMS testing.

The TMS component of the protocol proceeded as follows: (1) Identification of FDI hotspot location: TMS pulses were applied while the coil position was optimized so that the FDI motor hot spot could be identified. The corresponding coil position was marked on a scalp cap, and the cap position on the head was denoted with a mark on the forehead. (2) Determination of 1 mV MEP intensity: TMS pulses were usually started at ~55% of maximum stimulator output (% MSO), and the stimulation intensity was adjusted while MEPs were monitored and quantified online until the MEP amplitudes were as close as possible on average to 1 mV. Once this was accomplished, the software program was reset to collect the pre-test TMS block. (3) Pre-test TMS block: twenty-five MEPs were collected using the 1 mV stimulation intensity. (4) Five minutes of tDCS or SHAM stimulation: following the previous step, the TMS cap was removed, the tDCS electrode electrodes were placed on the head, and five minutes of either tDCS or SHAM stimulation was delivered. (5) Post-test TMS block: Immediately after the five minutes of stimulation elapsed in the previous step, three prepared investigators coordinated as quickly and as accurately as possible to remove the tDCS electrodes, reposition the TMS cap and coil, and begin the collection of the post-test TMS block (25 MEPS) as soon as possible using the same 1 mV stimulation intensity. At this time, the participant was instructed to maintain relaxation of the hand and to minimize overall movement. (6) Twenty-minute inter-stimulation period: one investigator set a 20 min time clock (at the end of step four) and enforced a 20 min interval between the end of the five-minute tDCS application and the subsequent start of the 20 min tDCS application period that was performed concurrent with the practice blocks of overhand throwing (see Figure 2).

The novel paradigm described above involved a five-minute tDCS application followed by a 20 min interval and was designed to address methodological issues related to MEP quantification before and after tDCS application. It was based on research findings by other research groups in studies that were focused entirely on the influence of different tDCS duration protocols on primary motor cortex excitability and are described briefly below. The paradigm developed for the purposes of this study was piloted in our laboratory for the current study and two other studies (unpublished, in progress). This was undertaken to ensure, as much as possible, that this protocol worked as envisioned.

The paradigm was developed based on three interrelated methodological considerations: (1) tDCS given for 3–5 min increases MEPs for a 3 to 5 min period after stimulation ceases [26,27,28]; (2) when a 20–30 min break is given before another application of tDCS, the same exact pattern of MEP increases occur as before. However, if a short break of only three to 10 min is given, inhibition occurs [26,27]; and (3) MEP increases due to tDCS can be abolished after muscle contractions (e.g., isometric contractions), movements such as walking, and following various other motor and even cognitive activities [29,30,31,32]. This can lead to MEP measurements after practice being meaningless (for a review of these issues, see Horvath et al. (2014) [30]). Thus, the novel paradigm above was designed to mitigate these problems while retaining the ability to quantify possible associations between the enhancements in MEP amplitudes and increases in motor learning [20,21,33]. However, it assumes that the second tDCS application had the same effects on primary motor cortex excitability effects as the first tDCS application [26,27].

#### 2.3.3. Practice Blocks

The practice blocks of overhand throwing trials were completed simultaneously with either tDCS or SHAM stimulation. This was conducted for a total practice and stimulation period of 20 min (Figure 2A). The practice block segment progressed as follows: (1) tDCS was applied for three minutes while subjects stood quietly before starting the first block of overhand throwing trials [24], (2) five blocks of overhand throwing were performed (10 trials per block) and were completed within the remaining 17 min of stimulation time. Each block took ~1 to 1.5 min to complete, and a two-minute rest interval was utilized between blocks, and (3) the stimulator kept running after the last block of overhand throws was completed (usually one to two minutes) until the 20 min stimulation period had elapsed. The investigator who programmed the stimulator for the practice blocks did not participate in the data collection or data analysis portions of the experiment.

#### 2.3.4. Post-Test Blocks

After the stimulation period and associated practice blocks had ended, participants were required to stand in place quietly while the now inert electrode montage remained on the head. Next, a five-minute rest period was enforced before performing the post-test block of overhand throwing 10 trials. The execution of the post-test blocks without simultaneous tDCS allowed for the calculation of the roles of online and offline learning effects on total motor learning when incorporated into calculations with the pre-tests that were also completed without stimulation (see Section 2.7 Statistical Analysis).

### 2.4. tDCS

A NeuroConn DC Stimulator Plus/MR delivered anodal tDCS at a current strength of 1 mA through a pair of rubber electrodes (5 cm × 7 cm) that were encased in sponges soaked with saline solution. The anode was placed over the FDI motor hotspot of the left primary motor cortex, and the cathode was placed over the contralateral supraorbital (M1-SO montage). The anode and cathode were secured in place by rubber straps. As described above, tDCS was delivered for a five-minute period between the TMS pre-test and post-test blocks and for a 20 min period during the practice blocks of overhand throws. Therefore, the two applications of tDCS had the same stimulation parameters other than two different durations. In the overhand throwing trials, the stimulation device was positioned in the backpack [24], whereas the stimulator was positioned on a small table behind the participant during MEP testing. SHAM stimulation was applied according to standard procedures [34], which involved the current being ramped up over 10 s, held constant at 1 mA for 30 s, and ramped down over 10 s.

### 2.5. Overhand Throwing Task

The overhand throwing task was executed in a manner identical to a previous study in our laboratory that used similar experimental methods [24], although it was a single-day study involving c-tDCS. Participants stood 6 m from a wall and behind a line marked on the floor. A sturdy board was mounted on the wall, and a printed poster covered with clear industrial tape was nailed to the board surface. The target (Figure 2B) was printed on the poster that displayed a very small 1 cm diameter “bull’s-eye” center. 

All participants threw a tennis ball in a manner similar to an overhand baseball throw using the right (dominant) arm. The instructions were to perform every throw as accurately as possible by attempting to hit the target center on each trial. An investigator covered the ball with red chalk before and halfway through each block of 10 trials. This allowed marks to be made that denoted the final endpoint position of the ball after hitting the target area. Thus, participants could use visual feedback of the endpoint of the ball relative to the target center after a trial to facilitate minimizing the distance of the error in subsequent trials. After each trial, that investigator retrieved the ball and gave it back to the participant to start the next trial while another investigator recorded the ball mark in the trial with a very small trial-numbered sticker. Participants stood quietly and rested after each trial block ended while three investigators used the rest interval to measure, record, and enter the endpoint *x* and *y* coordinates of the 10 stickers directly into a data file on a laptop computer. They also removed the stickers from the target area during the rest interval, and the process repeated for the following block of trials.

The overhand throwing task was always performed when wearing a tightly fitting small backpack with the tDCS device inside. The tDCS device was only turned on in the practice blocks (Figure 2) and, therefore, was not on during the test blocks. Crucially, however, the now inert electrode montage remained on the head of the participant and the device in the backpack so that the overhand throwing conditions were the same in all blocks. The arrangement of the tDCS device, montage, and backpack did not restrict overhand throwing performance [24]. Accordingly, the overhand throws were executed naturally in unconstrained conditions in three dimensions. Collectively, the overhand throwing task itself, the very small target size, and the long throwing distance were task details purposefully chosen within the lab space constraints to make certain that the motor task would be very challenging.

A three-dimensional overhand throwing task performed in a manner similar to a baseball throw was chosen as the motor practice task, as it is arguably one of the most complex human movements (for reviews, see [35,36]). First, overhand throwing is an unconstrained, ballistic, multi-joint skill that involves the prediction and exploitation of joint interaction torques [37,38,39,40,41]. Thus, the various rotations of multiple joints need to be timed to utilize the passive flow of kinetic energy between body segments to maximize the velocity of the end effector (hand). Accordingly, some of the highest joint angular velocities ever measured have occurred in overhand throwing [35]. Second, the modulation of the finger forces involved in the timing of the finger opening is extremely precise (1–2 milliseconds) in skilled throwers and is a major determinant of accuracy [42,43,44,45,46]. This is because even timing errors slightly greater than a few milliseconds can lead to ball releases along the hand path that result in large endpoint errors [43]. Third, the associated timing and coordination of multiple sets of agonist and antagonist muscles must be strictly regulated [37,44]. More specifically, overhand throwing is a whole-body movement that involves almost all the muscles of the body as the momentum from the lower extremities and trunk is transferred to the muscles of the arm and hand. Fourth, it is characterized by a large movement amplitude of the arm, usually performed at relatively high velocities, and involves an exceedingly large number of degrees of freedom. Therefore, the components that comprise the speed-accuracy trade-off (accuracy, movement velocity, and movement amplitude) are extremely high in overhand throwing. Taken together, it can easily be seen how overhand throwing is a much more complex task than isometric pinch grip tasks of the thumb and index finger or serial reaction time tasks of the fingers that have been used in a large proportion of previous tDCS studies [1].

### 2.6. Data Analysis

The endpoint error in the overhand throwing task was the primary dependent measure of interest, and the MEP amplitude evoked by TMS was the secondary dependent measure of interest. The dependent variables, age, laterality quotient, and 1 mV MEP intensity, were considered control measures. Endpoint error is the best overall measure of endpoint accuracy and was calculated in accordance with prior studies [24,47,48,49]. Briefly, the Pythagorean Theorem was used to employed to calculate the shortest absolute distance between the target center’s *x*, *y* coordinates and the ball’s final endpoint *x*, *y* coordinates (see [50] for details of the calculations). The *x and y* coordinates of the ball’s endpoint were entered directly into a custom-written script in Microsoft Excel, which calculated endpoint errors for each trial and block of trials. The average of the 10 trials in each block of overhand throwing was denoted as the final endpoint error values and used for analysis. MEP data were collected using a customized script written in Signal software (Cambridge Electronic Design, Cambridge, UK) and further analyzed offline using another custom-written script in Signal. MEP size was quantified as the peak-to-peak amplitude for each individual MEP, and the average of the 25 MEPS evoked in each test block was used for analysis. Finally, the control measures of average age, laterality quotient, and 1 mV MEP intensity (three-day grand average) were quantified for each of the two groups.

### 2.7. Statistical Analysis

Endpoint error was analyzed using statistical methodologies employed in previous three to five-day tDCS studies by other research groups [12,13,51] but also shared similarities with our previous studies [24,47,49]. The statistical analysis of endpoint error involved three steps: (1) Endpoint error from only the test blocks was analyzed with a 2 *Group* (tDCS, SHAM) × 3 *Day* (1, 2, 3) × 2 *Test* (pre-test, post-test) three-way mixed ANOVA. This analysis used the test block endpoint error only because they were executed without concurrent stimulation. Furthermore, this facilitated comparison of the results to previous three to five-day tDCS studies [12,13,51]; (2) endpoint error from all the test blocks and practice blocks for each day were averaged and were analyzed with a two-way mixed ANOVA: 2 *Group* (tDCS, SHAM) × 3 *Day* (1, 2, 3). Thus, this second analysis used the average endpoint error value obtained from all seven blocks combined (two tests and five practice blocks) on each day. This complemented the first analysis since our pilot data and a previous single-day study [24], as well as the present study, and all had many individual participant instances where endpoint error in a test block could be rather different from some practice blocks. Accordingly, this analysis could potentially more accurately represent overall performance for each day; (3) unpaired two-tailed *t*-tests were used to compare the online, offline, and total learning effects between the two groups (see Cantarero et al. [40] for details of the calculations).

MEP amplitude was analyzed with a three-way mixed ANOVA: 2 *Group* (tDCS, SHAM) × 3 *Day* (1, 2, 3) × 2 *Test* (pre-test, post-test). Bivariate linear regression analyses were also utilized to analyze the correlations between MEP amplitude changes between the TMS pre-tests and post-tests and endpoint error changes between the pre-test and post-test blocks involving overhand throwing. These correlations were performed separately for each of the three days and only for the tDCS group. The control measures of age, laterality quotient, and 1 mV MEP intensity were compared between groups with separate unpaired two-tailed *t*-tests. Post hoc comparisons using Bonferroni adjustment for multiple comparisons were performed to locate where significant differences occurred between pairs of means in all of the ANOVAs described above. The significance level was α < 0.05 for all analyses, and the data are shown as means ± standard errors in the figures.

## 3. Results

### 3.1. Endpoint Error

**Figure 3:**
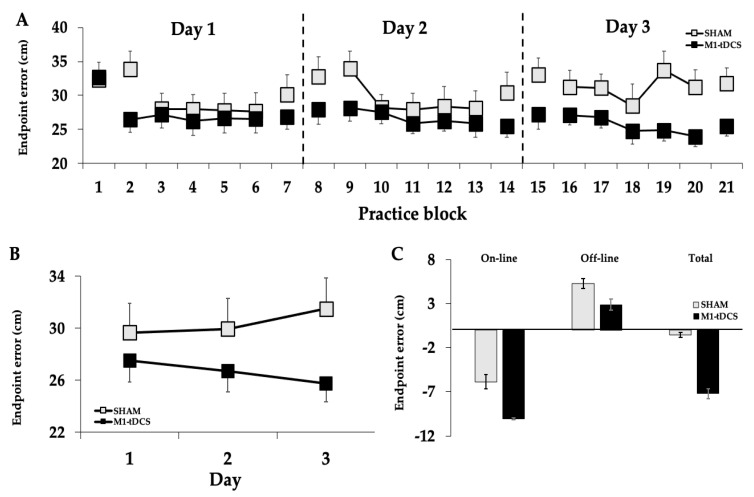
Endpoint error in overhand throwing for the tDCS and SHAM groups. (**A**) The endpoint error as a function of the trial block number for all three days. The endpoint error declined across the test blocks for the three days of practice but only for the tDCS group (*Group* × *Day* interaction; *p* = 0.034); (**B**) endpoint error averaged for all seven daily trial blocks. The endpoint error declined across the test blocks for the three days of practice but only for the tDCS group (*Group* × *Day* interaction; *p* = 0.017); (**C**) the online (*p* = 0.343) and offline learning (*p* = 0.418) were similar for the two groups. However, the combined online and offline effects resulted in significantly greater total learning (*p* = 0.047) for the tDCS group compared to the SHAM group.

The differences in motor learning between the groups were compared across the practice days and test blocks with a 2 *Group* (tDCS, SHAM) × 3 *Day* (1, 2, 3) × 2 *Test* (pre-test, post-test) ANOVA. There was a significant *Group* × *Day* interaction (*p* = 0.034, *η*^2^ = 0.074; Figure 3A), and post hoc analyses of the interaction indicated that endpoint error when collapsed across *Test* was significantly lower in the tDCS compared to the SHAM group on day 3 (*p* = 0.043), but not day 1 (*p* = 0.654) or day 2 (*p* = 0.125). There was also a significant main effect for *Test* (*p* < 0.001, *η*^2^ = 0.23), which indicated that endpoint error was lower in the post-tests compared to the pre-tests. The main effect for *Group* (*p* = 0.165, *η*^2^ = 0.043), main effect for *Day* (*p* = 0.274, *η*^2^ = 0.029), *Group* × *Test* interaction (*p* = 0.343, *η*^2^ = 0.02), *Test* × *Day* interaction (*p* = 0.348, *η*^2^ = 0.024), and *Group* × *Day* × *Test* interaction (*p* = 0.533, *η*^2^ = 0.014) were all non-statistically significant.

The differences in motor learning between groups were also compared across the practice days using average endpoint error data when collapsed across all the practice and test blocks with a 2 *Group* (tDCS, SHAM) × 3 *Day* (1, 2, 3) mixed ANOVA. There was a significant *Group* × *Day* interaction (*p* = 0.017, *η*^2^ = 0.088; Figure 3B), and post hoc analyses of the interaction indicated that endpoint error was significantly lower in the tDCS compared to the SHAM group on day 3 (*p* = 0.042), but not day 1 (*p* = 0.441) or day 2 (*p* = 0.26). The main effect for *Day* (*p* = 0.887, *η*^2^ = 0.003) and the main effect for *Group* (*p* = 0.174, *η*^2^ = 0.042) were both non-statistically significant.

Due to the fact that the ANOVAs revealed significant *Group* × *Day* interactions for total motor learning, a series of separate unpaired two-tailed *t*-tests were used to compare online, offline, and total learning effects between groups. The analyses revealed that the online and offline effects were non-statistically significant between the tDCS and SHAM groups (*p* = 0.343 and *p* = 0.418, respectively). However, the total learning effect was significantly (*p* = 0.047; Figure 3C) greater for the tDCS group compared to the SHAM group.

### 3.2. MEP Amplitude

**Figure 4:**
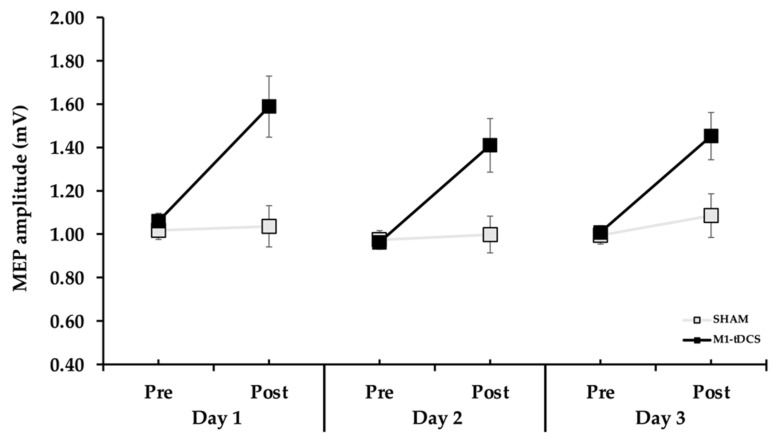
MEP amplitude for the tDCS and SHAM groups in the TMS pre-tests and post-tests for the three days. The MEP amplitude was significantly increased between the TMS pre-tests and post-tests on all three days for the tDCS group (*p* < 0.001) but not for the SHAM group.

Primary motor cortex excitability differences between groups were compared across practice days and test blocks with a 2 *Group* (tDCS, SHAM) × 3 *Day* (1, 2, 3) × 2 *Test* (pre-test, post-test) ANOVA. There was a significant *Group* × *Test* interaction (*p* < 0.001, *η*^2^ = 0.286; Figure 4), and post hoc analyses of the interaction indicated that MEP amplitude, when collapsed across *Day,* was significantly greater in the tDCS compared to the SHAM group in the post-tests (*p* = 0.034), but not the pre-tests (*p* = 0.104). Accordingly, there was a significant main effect for *Test* (*p* < 0.001, *η*^2^ = 0.370), which indicated that MEP amplitude was higher in the post-tests compared to the pre-tests. Finally, there was a significant main effect for *Group* (*p* < 0.001, *η*^2^ = 0.264)*,* which indicated that MEP amplitude was higher in the tDCS compared to the SHAM group. The main effect for *Day* (*p* = 0.271, *η*^2^ = 0.029), *Group* × *Day* interaction (*p* = 0.566, *η*^2^ = 0.013), *Test* × *Day* interaction (*p* = 0.930, *η*^2^ = 0.002), and *Group* × *Day* × *Test* interaction (*p* = 0.744, *η*^2^ = 0.007) were all non-statistically significant.

### 3.3. Associations between Increases in MEPs and Increases in Endpoint Accuracy

**Figure 5:**
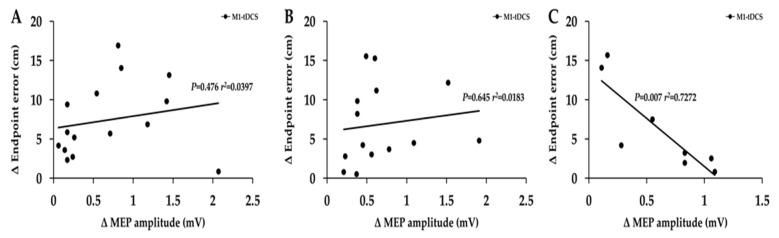
Associations between increases in MEP amplitude and increases in endpoint accuracy for the tDCS group. (**A**–**C**) The absolute change (increase) in endpoint accuracy (decrease in endpoint error) was not associated with the absolute change (increase) in MEP amplitude for the participants in the tDCS group that displayed both increases in endpoint accuracy and MEP amplitude.

Bivariate linear regressions were performed for each of the three days. Importantly, only participants in the tDCS group who had exhibited both increases in MEP amplitude and endpoint accuracy were included in these analyses. The results revealed that on days 1 and 2, the associations between the changes in MEP amplitudes change in endpoint error (endpoint accuracy) for the tDCS group were all non-statistically significant (*p* = 0.476 and *p* = 0.645, respectively), supported by the *r*^2^ values (0.04 and 0.18, respectively) as indicated in Figure 5A,B. On day 3, the analysis revealed that the associations were significant (*p* = 0.007, *r*^2^ = 0.727); however, the r value was negative (−0.853).

### 3.4. Control Measures

A series of separate unpaired *t*-tests revealed that the between-group differences for age and laterality quotient were non-statistically significant (*p* = 0.136 and *p* = 0.718, respectively). The average 1 mV MEP intensity (% MSO) for the whole sample was 51.5%. An unpaired t-test revealed that the 1 mV MEP intensity (% MSO) was not significantly (*p* = 0.832) different between the two groups (tDCS group average: 51.1%; SHAM group average: 51.8%).

## 4. Discussion

The primary purpose of the current study was to examine the influence of tDCS on motor learning over multiple days in a complex overhand throwing task in young adults, whereas the secondary purpose was to examine the association between tDCS induced increases in cortical excitability and the magnitude of motor learning. This study produced four main findings. First, endpoint error significantly decreased over the three days of practice in the tDCS group but not in the SHAM group. Second, the decreases in endpoint error were due to both online and offline effects in the tDCS group. Third, MEP amplitude was significantly increased in the tDCS group but not in the SHAM group. Fourth, the increases in MEP amplitude in the tDCS group were not positively associated with the degree of improvements in motor learning. Taken together, these findings indicate that M1-tDCS applied over three consecutive days can improve motor learning in a complex motor task in young adults, but these improvements are not associated with tDCS-induced changes in primary motor cortex excitability.

### 4.1. Influence of M1-tDCS on Motor Learning

Motor learning refers to a relatively long-term improvement in motor performance due to extensive practice. It is usually quantified at a minimum of several hours or one-day following practice. In contrast, motor skill acquisition refers to a relatively short-term change in motor skill measured during a single practice session or within a couple of hours following practice. In addition, the physiological mechanisms and adjustments underlying the motor skill and motor learning processes over these distinct time periods in normal practice circumstances are different in some respects [52]. Similarly, the physiological mechanisms mediating the positive effects of tDCS on motor skill learning during short-term versus long-term motor skill learning are likely realized through some common but also some distinct mechanisms [1]. Regardless of the exact physiological mechanisms, extensive research over many years has shown that the primary motor cortex is one of the most important, if not the predominant, brain regions responsible for the improvements in motor learning with practice [1,53,54,55]. Accordingly, this is one major reason that studies utilizing tDCS and related non-invasive brain stimulation techniques most commonly target the primary motor cortex when attempting to improve motor learning, although they have primarily involved relatively simple motor tasks.

The current study was the first to our knowledge that has investigated the influence of tDCS applied to the primary motor cortex on motor learning over multiple days in a complex motor task involving whole-body coordination. It was hypothesized that the tDCS group would display greater total motor learning over the course of the three days of practice and stimulation compared to the SHAM group (practice alone). Consistent with this hypothesis, endpoint error slowly and progressively decreased with practice in the tDCS group by approximately 22% by the end of day 3. In contrast, the SHAM group experienced an improvement in endpoint error of only about 2% in this difficult motor task at the end of day 3. These differences in total motor learning, however, were not accompanied by statistically significant between-group differences in online or offline learning (Figure 3C). This was because both the online and offline effects equally contributed to the greater total motor learning in the tDCS group (see below). The greater motor learning realized by the tDCS group was also not due to the increases in MEP amplitude found only in the tDCS group because the increases were not correlated with the amount of motor learning. Finally, potential factors such as the baseline skill level, age of the participants, transcranial magnetic stimulation (TMS) stimulation intensity to evoke a 1 mV MEP [56], and degree of right-handedness, which could have possibly contributed to the between-group differences, cannot explain the present findings as these factors were almost exactly the same between groups.

The results of the present study are congruent with most of the prior single-session tDCS studies by other research groups [1] that utilized simple motor tasks. The findings are also in agreement with a previous single-session study, albeit involving anodal cerebellar tDCS, performed in our lab that used the exact same overhand throwing task [24]. In that study, the reduction in endpoint error was greater for the cerebellar tDCS group (~19.5%) compared to the SHAM group (~6.5%) at the end of the practice session. However, the results of the current study are best analyzed in the context of multi-day studies that also applied tDCS to the primary motor cortex.

Overall, the current outcomes are also generally in accordance with two previous M1-tDCS studies that utilized the SVIPT of the thumb and index fingers but also displayed a few nuanced differences. First, the magnitude of difference between the tDCS and SHAM groups in total motor learning appears to be less (~22%) compared to at least 40% between-group differences that the figures in the studies by Reis and colleagues [12,13] appear to show. Second, the between-group differences in online and offline effects were not statistically different between the tDCS and SHAM groups in the current study, whereas Reis et al. reported significant between-group differences in offline effects. Therefore, the greater total learning in the present study had to be due to the online and offline effects, both contributing to the significant total learning differences between groups, although each individual did not reach statistical significance when compared between the two groups. Accordingly, the average decrease in endpoint error for the online effects was ~−5.86 cm for the SHAM group versus −10.08 cm for the tDCS group, which is almost a 50% difference. For the offline effects, the endpoint error increase was 5.3 cm for the SHAM group versus 2.88 cm for the tDCS group, which again is almost a 50% difference. Note that these increases in offline learning represent a regression from the skill levels achieved at the end of the previous day. Thus, the tDCS group had less of a regression day to day along with greater online learning day to day. Third, the SVIPT studies appeared to show that most of the differences between groups were realized within the first few blocks of trials and that this difference was mainly maintained over the course of the next three to five days. In contrast, the results of the current study did show large between-group differences in the first tDCS block on the first day, but this difference was quickly made up by the SHAM group. Subsequently, the tDCS group improved overall motor learning through slow, progressive improvements across the trial blocks within and between the three days. Nonetheless, the current findings are in general agreement and extend previous multi-day tDCS involving simple motor tasks to more complex motor tasks. However, tDCS effects on motor learning, at least in this complex motor task, do not appear to be as great in magnitude, as consistent, or mediated primarily through offline effects compared to prior research on simpler tasks.

### 4.2. The Influence of tDCS on Primary Motor Cortex Excitability Measured by Transcranial Magnetic Stimulation

Transcranial magnetic stimulation (TMS) is a non-invasive brain stimulation technique that, in single or paired-pulse mode, can be used to assess corticospinal excitability at rest and during muscle activity. A TMS pulse applied at a stimulation intensity above the threshold to the primary motor cortex elicits an electromyographic (EMG) response termed the motor-evoked potential (MEP). The MEP is one of the most common measurements in the neurophysiology of movement studies and provides a global measure of net corticospinal excitability. Thus, one of the primary experimental uses of the MEP is to quantify physiological changes in the primary motor cortex before and after some intervention. Accordingly, in the present study, MEPs in response to single-pulse TMS were recorded before and after the application of anodal tDCS to measure changes in primary motor cortex excitability.

Anodal tDCS delivered to the primary motor cortex usually increases both motor skill and primary motor cortex excitability as measured by MEP amplitude evoked by single-pulse TMS. These are common observations that have been reported in studies that measured only one of these variables or in studies that have measured both in the same study. Furthermore, the increases in motor skills and MEPS were shown to be positively correlated in some initial studies [20,21]. Based on these results and on classic studies that observed enhanced MEPs in task-specific muscles following deliberate skill practice (no tDCS involved) [6], it was initially assumed that the increases in primary motor cortex excitability induced by tDCS were at least partially responsible for motor skill improvements that surpassed practice alone. However, findings from recent studies and review articles [30,33,57,58] have cast doubt on whether the concurrent increases in motor performance and MEP amplitudes are strongly associated and mechanistic linked or even if the increased MEP values in these instances have any functional relevance. Therefore, the secondary purpose of this study was to examine if increases in primary motor cortex excitability due to tDCS, if they occurred, would be positively associated with the magnitude of motor learning exhibited by the participants.

For MEP amplitude, the major overall finding was that tDCS significantly increased MEP amplitude from the pre-tests to the post-tests on all three days (Figure 4) but was essentially unchanged in the SHAM group. These results were expected and in line with the majority [59], although not universally supported by all previous studies on the topic [30,58,60]. More specifically, the MEP amplitude increases were about 47% when averaged over the three days for the tDCS group compared with the non-significant increase of about 5% in the SHAM group. This degree of increase is at the high end of the approximate 20–50% range reported in most of the early tDCS studies on the topic [58]. A more recent review found the average MEP increase across all anodal tDCS studies to be 28% [59]. Furthermore, the average percentage of participants in the tDCS group who displayed a MEP increase on a given day was 74%, whereas the corresponding value in the SHAM group was 46%. In regard to the associations between MEP amplitude changes and endpoint accuracy changes, the results demonstrated a complete lack of significant positive associations even though the analyses were confined to only participants in the tDCS group who exhibited an increase in both measures on a given day. Accordingly, the *r*^2^ values were exceedingly small on days 1–2, and the *r* value was even negative for day 3 [Figure 5A–C], which further implies that MEP increases following tDCS have relatively little functional relevance to motor skill learning. These results are in contrast to two previous studies that involved small sample sizes in patient populations [20,21] but are consistent with a very extensive study with a very high sample size [33]. This study found that MEP increases elicited by several forms of non-invasive brain stimulation, including tDCS, were not associated with the amount of motor learning achieved by participants across three of the most common motor tasks used in tDCS studies [33].

The exact physiological mechanisms underlying the lack of association between increases in motor learning and MEP amplitude induced by tDCS are not known precisely and were not investigated in the most extensive prior study on the topic [33]. Furthermore, the examination of possible mechanisms was well outside the scope of the current study. Therefore, we can only broadly speculate on the major reasons why increases in MEP and motor learning were not associated in most previous studies as well as the current one. First, the MEP has many inherent limitations for use in interpreting changes in primary motor cortex physiology, especially relative to complex motor behavior. As a result, changes in MEPs can oftentimes have no causal relationship to motor performance (for review, see [57]). Second, MEP changes were only measured in the FDI muscle of the throwing hand. Although this muscle is likely important in the crucial aspect of the timing of finger release, overall overhand throwing accuracy most certainly results from the coordinated activation and timing of numerous muscles. Third, measures of MEP changes at rest before and after tDCS may have little relevance to possible MEP changes measured during muscle activation. Certainly, the issue of the disconnect between increases in MEP and motor learning is challenging and will require further investigation in studies that combine multiple physiological measures with a motor learning paradigm.

### 4.3. Clinical Implications

The findings of the current study could have clinical implications in numerous populations if long-term tDCS application could elicit similar or even greater complex motor task improvements in neurological disorders than those observed in healthy young adults; for example, children with autism spectrum disorder [61,62] and cerebral palsy [63]. Accordingly, tDCS has generally been found to improve motor and cognitive performance and learning in these two child populations [64,65,66], although all these studies have employed rather simple motor tasks compared to the present study. This study could have even greater implications for motor disorders in adults as tDCS has been studied in almost every movement disorder, with Parkinson’s disease, stroke, and Multiple Sclerosis (MS) having received the most attention. For instance, the balance of tDCS studies in PD have shown a positive effect on various aspects of motor performance [9,67], but almost all of these studies also involved simple motor tasks. In addition, numerous studies have found that tDCS is efficacious for motor recovery after stroke [68], especially when combined with other rehabilitation methods [69]. Finally, the preponderance of studies conducted in MS has shown significant effects due to tDCS, with the strongest findings being observed in measurements involving cognitive and physical fatigue [70,71]. In summary, the vast majority of tDCS motor skill and learning studies in every population have involved simple motor tasks, and the findings of the current study suggest that tDCS has the potential to also improve motor learning in complex tasks in populations other than healthy young adults, although perhaps to the same extent as in simple motor tasks.

### 4.4. Limitations and Future Directions

Despite the rather straightforward findings that tDCS improved motor learning to a greater degree than SHAM stimulation, this study had some limitations that should be recognized. First, the study lacked long time-scale motor learning retention tests as it only contained post-test blocks performed 5 min after stimulation each day. It would have been ideal to have participants return for follow-up testing in the days, weeks, or even months after stimulation and practice had ended, as conducted previously [13]. Second, the current study only contained one behavioral measure (endpoint error) and one physiological measure (MEPs). The inclusion of other physiological measures, such as EEG concurrent with the TMS recordings, along with three-dimensional kinematics during the overhand throwing, would have been able to give much more precise information on the mechanisms underlying the greater motor learning induced by tDCS. Third, it is possible that even greater effects on motor learning due to tDCS could have been observed if the number of stimulation days had been higher (e.g., 5 days to a month). Fourth, this study did not have any transfer of motor learning tasks to determine if the increases in motor learning resulting from tDCS could be generalized to other tasks that were not practiced [49]. Fifth, the sample size could be viewed as a final limitation. Although the sample size per group of 23 was much higher than the average motor skill study involving tDCS of about 13 per group (see tables of Buch et al. [1]), the sample size is always a limitation in neuroscience studies [72,73]. All of these limitations will have to be addressed in subsequent studies.

Future studies should also investigate the interrelated issues of individualization of tDCS application, different stimulation parameters (e.g., montage, current strength, type of stimulation), population studied, and other types of brain stimulation. In regard to individualization, accumulating evidence suggests that the interindividual susceptibility and the variability of performance responses to tDCS could result from some combination of differences in anatomical and physiological factors (for reviews, see [74,75]). This has arguably been better studied when tDCS is applied to the cerebellum [76,77], but few studies exist that have been able to take into account and use even a few individual factors to achieve more targeted stimulation [77,78]. Nonetheless, neuronavigation using fMRI approaches to target electrode placement and modulating current strength levels based on skull and cerebrospinal fluid thickness have begun to be utilized [74] and would be valuable future avenues of research to optimize the effects of tDCS. Furthermore, stimulation parameters other than the common and effective M1-SO montage with a 1 mA current strength applied for 20 min could be more effective. For example, Naro and colleagues [79] demonstrated that both bihemispheric and dual double-source tDCS improved motor learning to a greater extent than the M1-SO montage. Similarly, high-definition tDCS and/or 4 mA current strengths could also lead to better results in various motor tasks [78,80]. In addition, more studies using complex motor tasks in other populations, such as expert performers or individuals with motor disorders, where either ceiling or floor effects are likely to influence tDCS performance responses, should be investigated in future studies. Finally, other brain stimulation methods, such as repetitive transcranial magnetic stimulation (rTMS), have shown promise in improving motor performance, but practical limitations, as well as inconsistent or mild positive effects, limit its applicability [81]. However, tDCS may be a more effective form of non-invasive brain stimulation compared to rTMS as, overall, more studies have reported increases in motor performance with tDCS compared to rTMS, which was the primary reason tDCS was used in the current. In addition, tDCS offers several important practical advantages over rTMS, such as portability, safety, ease of administration, ability to be delivered during motor activities, a superior ability to blind subjects with SHAM stimulation, and low cost (as low as $400 vs. $20,000–100,000 for rTMS) [81].

## 5. Conclusions

The main finding was that endpoint error significantly decreased over the three days of practice in the tDCS group but not in the SHAM group. Thus, M1-tDCS was able to improve motor learning in this complex motor task to a greater extent than practice alone in healthy young adults. The greater total motor learning exhibited by the tDCS group was accomplished through both greater online and offline learning effects. tDCS also significantly increased primary motor cortex excitability, but these excitability increases were not significantly positively associated with the amount of motor learning. The results confirm and extend the overall tDCS motor learning literature in simple motor tasks. However, the magnitude of motor learning participants displayed in this complex motor task did not seem to be as high as that previous multi-day M1-tDCS studies involving a pinch grip task [12,13].

## Data Availability

The data presented in this study are available on request from the corresponding author.
